# Templated microbiology comments with candiduria to enhance antimicrobial stewardship

**DOI:** 10.1017/ash.2022.292

**Published:** 2022-09-14

**Authors:** Weston R. Schartz, Nicholas Bennett, Laura Aragon, Kevin Kennedy, Austin Wilson, Sarah Boyd, Matthew Humphrey, Cynthia Essmyer

**Affiliations:** 1 Department of Pharmacy, Saint Luke’s Health System, Kansas City, Missouri; 2 Antimicrobial and Diagnostic Advisement Program, Saint Luke’s Health System, Kansas City, Missouri; 3 Department of Biostatistics, Saint Luke’s Health System, Kansas City, Missouri; 4 Department of Pharmacy, The University of Texas MD Anderson Cancer Center, Houston, Texas; 5 Department of Infectious Diseases, Saint Luke’s Health System, Kansas City, Missouri; 6 Department of Microbiology, Saint Luke’s Health System, Kansas City, Missouri

## Abstract

**Objective:**

To evaluate the effect of templated microbiology reporting comments on antifungal utilization in patients with candiduria.

**Design:**

In this retrospective, quasi-experimental study, we evaluated a preimplementation cohort (June 2018–January 2019) compared with a postimplementation cohort (June 2019–January 2020).

**Setting:**

A multisite health system including 1 academic hospital and 4 community hospitals.

**Patients:**

Patients were aged ≥18 years, were hospitalized, and had candiduria documented at least once during their admission. The study included 156 patients in the preimplementation period and 141 patients in the postimplementation period.

**Methods:**

In June 2019, Saint Luke’s Health System implemented the use of templated comments for urine cultures with *Candida* spp growth. When *Candida* is isolated, the following comment appears in the microbiology result section: “In the absence of symptoms, *Candida* is generally considered normal flora. No therapy indicated unless high risk (pregnant, neonate, or neutropenic) or undergoing urologic procedure. If Foley catheter present, remove or replace when able.” The primary outcome was rate of antifungal prescribing.

**Results:**

Antifungal administration within 72 hours of a culture identifying a *Candida* spp occurred in 75 patients in the preimplementation group and 48 patients in the postimplementation group (48.1% vs 34.0%; *P* = .02). We did not detect a difference between groups in antifungal administration between 73 and 240 hours (1.3% vs 3.5%; *P* = .26), nor did we detect a difference in median antifungal duration (4 vs 3 days; *P* = .43).

**Conclusion:**

Using a templated comment with urine cultures reduced antifungal prescription rates in hospitalized patients with candiduria. This strategy is a low-resource technique to improve antimicrobial stewardship.

Antimicrobial stewardship programs (ASPs) aim to minimize antimicrobial resistance, improve patient outcomes, reduce untoward adverse events, and optimize resource utilization through the promotion of evidence-based practice.^1^ It has been estimated that up to 50% of antimicrobial use is inappropriate.^2^ Implementing behavioral interventions to guide healthcare providers in treating infections has been shown to reduce the use of inappropriate antibiotics.^2–5^ One such intervention comprised passive education utilizing microbiology results, sometimes referred to as nudging, which has been shown to be an effective vehicle to promote stewardship.^6–8^ Previous studies have leveraged microbiology comments to positively impact antibiotic prescribing for patients with pneumonia.^3,9^ This passive stewardship technique is intended to educate and influence while promoting medical provider autonomy in making treatment decisions.

Drug-resistant fungal infections are increasing in the United States.^10,11^ It has been estimated that 10%–15% of hospital-acquired urinary tract infections are caused by *Candida* spp.^12^ Generally, asymptomatic growth of *Candida* in urine cultures is considered colonization, not infectious.^12,13^ Treatment of asymptomatic *Candida* infections contributes to antifungal overuse and, potentially, resistance.^14^ Clinical practice guidelines recommend against the treatment of asymptomatic candiduria unless the patient is considered high risk.^13^ High-risk patients include neutropenic patients, very low-birth-weight infants (<1,500 g), and patients who undergo urologic procedures.^13^ Although guidelines recommend against treatment, the overtreatment of asymptomatic candiduria continues to be a problem in hospitalized patients.^15,16^

In June 2019, Saint Luke’s Health System developed and implemented templated comments for urine cultures that had *Candida* growth. The goal was to passively influence antifungal prescribing and to subsequently assess the impact of templated microbiology reporting comments in patients with candiduria.

## Methods

### Study design

This quasi-experimental multisite study was conducted at 5 hospitals within the Saint Luke’s Health System, Kansas City, Missouri. Patients were included in the study if they were aged ≥18 years, were hospitalized, and had a urine culture with *Candida* spp growth during admission. Patients were excluded if they received a systemic antifungal within 7 days prior to the culture result, if the urine culture was polymicrobial, or if they had an active suspected or confirmed nonurinary fungal infection. Patients with urinary *Candida* growth in the preimplementation cohort (June 2018–January 2019) were compared with those in a postimplementation cohort (June 2019–January 2020).

We evaluated the use of antifungal medications in patients with urine cultures growing *Candida* spp, according to electronic medical record (EMR) documentation. When *Candida* was isolated in the urine, the following comment appeared on the culture result: “In the absence of symptoms, *Candida* is generally considered normal flora. No therapy indicated unless high risk (pregnant, neonate or neutropenic) or undergoing urologic procedure. If Foley catheter present, remove or replace when able.” Only the first positive culture result per patient encounter was included.

Baseline characteristics were collected the day the urine culture was identified with *Candida* spp. The Saint Luke’s Health System’s ASP performed daily prospective audits with feedback during the pre- and postimplementation periods, and their practice remained unchanged during this time. Data were collected from the EMR for all patients (Epic, Verona, WI). The study received a waiver of approval from the institutional review board.

### Study outcomes

The primary outcome was the rate of antifungal administration within 72 hours after culture results showed *Candida* spp growth in the urine. The 72-hour time frame was chosen to give the provider adequate time to evaluate the urine culture result and to determine need for treatment. Secondary outcomes included duration of therapy and rate of antifungals prescribed within 73–240 hours after culture result. The 73–240-hour time frame was chosen to assess the safety of implementing the templated comment due to concerns that the comment may inadvertently cause providers to avoid treatment, when in fact, it may have been necessary.

### Statistical analysis

Baseline characteristics and outcomes with categorical variables were assessed with the Fisher exact test. Continuous variables were assessed with either the Student *t* test or the Wilcoxon rank-sum test for normally distributed and nonnormally distributed variables, respectively. Two-tailed statistical tests were utilized, with the significance level set at *P* < .05. Logistic regression was performed for the primary outcome to account for baseline differences between groups. This model was not parsimonious or predictive; rather, we aimed to adjust for potential confounding variables. Statistical analysis was completed using Stata version 14.2 software (StataCorp, College Station, TX).

## Results

The study included 297 patients between the 2 groups: 156 in the preimplementation group and 141 in the postimplementation group. Baseline characteristics were generally similar between groups (Table 1). Notable differences included a greater occurrence of the following in the postimplementation group: peripheral vascular disease (21.3% vs 7.1%; *P <* .01), myocardial infarction (24.8% vs 12.8%; *P* = .01), nephrolithiasis (19.1% vs 9.0%; *P* = .01), urinary stents (11.3% vs 2.6%; *P* < .01), and acute kidney injury (67.4% vs 46.2%; *P* < .01). The species most often isolated in the urine cultures of both groups was *Candida albicans* (Table 2).

**Table 1.** Baseline Characteristics

Variable	Preimplementation Group (n = 156)	Postimplementation Group (n = 141)	*P* Value
Age, mean y (SD)	69.3 (15.3)	69.8 (15.5)	.79
Sex, female, no. (%)	99 (63.5)	102 (72.3)	.11
**Race, no. (%)**			.29
African American	27 (17.3)	30 (21.3)	
Asian	1 (0.6)	0 (0.0)	
Hispanic or Latino	3 (1.9)	0 (0.0)	
White	122 (78.2)	107 (75.9)	
Pacific Islander	1 (0.6)	0 (0.0)	
Other/Unknown	2 (1.3)	4 (2.4)	
Weight, mean kg (SD)	83.7 (26.4)	85.0 (26)	.68
Height, mean inches (SD)	66.3 (4.0)	65.4 (3.8)	.04
Body mass index, mean (SD)	29.4 (8.6)	30.8 (8.9)	.19
Length of stay, median d (IQR)	9.0 (5.0, 19.0)	9.0 (5.0, 17.0)	.73
Acute kidney injury, no. (%)	72 (46.2)	95 (67.4)	<.01
Myocardial infarction, no. (%)	20 (12.8)	35 (24.8)	<.01
Chronic heart failure, no. (%)	59 (37.8)	62 (44.0)	.29
Peripheral vascular disease, no. (%)	11 (7.1)	30 (21.3)	<.01
Cerebrovascular accident or transient ischemic attack, no. (%)	31 (20.1)	28 (19.9)	1.00
Dementia, no. (%)	14 (9.0)	23 (16.3)	.08
Chronic obstructive pulmonary disease, no. (%)	30 (19.4)	35 (24.8)	.26
Connective tissue disease, no. (%)	5 (3.2)	8 (5.7)	.40
Peptic ulcer disease, no. (%)	5 (3.2)	8 (5.7)	.40
Chronic liver disease, no. (%)	6 (3.8)	9 (6.4)	.43
Hemiplegia, no. (%)	25 (16.0)	25 (17.7)	.76
Chronic kidney disease, no. (%)	91 (58.3)	104 (73.8)	.07
Hemodialysis, no. (%)	13 (8.3)	10 (7.1)	.83
Nephrolithiasis, no. (%)	14 (9.0)	27 (19.1)	.01
Urinary Stent, no. (%)	4 (2.6)	16 (11.3)	<.01
Instrumentation of urinary tract at time of culture collection, no. (%)	4 (2.6)	7 (5.0)	.36
Renal transplant, no. (%)	0 (0.0)	3 (2.1)	.11
Diabetes mellitus, no. (%)	80 (51.3)	87 (61.7)	.08
Malignancy, no. (%)	18 (11.5)	28 (19.9)	.06
Acute immune deficiency syndrome, no. (%)	0 (0.0)	1 (0.7)	.48
Leukemia, no. (%)	3 (1.9)	3 (2.1)	1.00
Lymphoma, no. (%)	4 (2.6)	1 (0.7)	.37
Serum creatinine, mean mg/dL (SD)	1.73 (1.49)	1.73 (1.32)	.99
White blood cell count, mean ×10^9^/L (SD)	11.7 (6.2)	13.9 (14.9)	.08
Charlson comorbidity index, mean (SD)	6.6 (3.1)	7.3 (2.8)	.046

Note. SD, standard deviation; IQR, interquartile range.

**Table 2.** *Candida* spp

Species	Preimplementation Group (n = 156), No. (%)	Postimplementation Group (n = 141), No. (%)	*P* Value
*Candida albicans*	92 (59.0)	75 (53.2)	.83
*C. dubliniensis*	1 (0.6)	3 (2.1)	
*C. glabrata*	35 (22.4)	27 (19.1)	
*C. kefyr*	3 (1.95)	3 (2.1)	
*C. krusei*	3 (1.95)	4 (2.8)	
*C. lusitaniae*	4 (2.6)	3 (2.1)	
*C.orthopsilosis*	1 (0.6)	1 (0.7)	
*C. parapsilosis*	4 (2.6)	4 (2.8)	
*C.tropicalis*	12 (7.7)	19 (13.5)	
*C. utilis*	0 (0.0)	1 (0.7)	
*Candida* spp not specified	1 (0.6)	1 (0.7)	

**Table 3.** Primary and Secondary Outcomes

Outcome	Preimplementation Group (n=156)	Postimplementation Group (n=141)	*P* Value
Antifungal administered within 72 h of *Candida* result; no. (%)	75 (48.1)	48 (34.0)	.02
Antifungal administered within 73–240 h *Candida* result; no. (%)	2 (1.3)	5 (3.5)	.26
Antifungal duration, median d (IQR)	4.0 (1–7)	3.0 (1–6)	.43

Note. IQR, interquartile range.

The primary outcome of antifungal administration within 72 hours was significantly lower in the postimplementation group (48.1% vs 34.0%; *P* = .02). We did not detect a difference for the secondary outcome of patients requiring antifungal administration within 73–240 hours after microbiology results were available (1.3% vs 3.5%; *P* = .26). Two patients in the study were diagnosed with candidemia later in their hospitalization, with 1 occurring in each group. There was also no difference in mean antifungal duration (4 vs 3 days; *P* = .43). After multivariable adjustment on characteristics that differed significantly between groups (ie, height, myocardial infarction, peripheral vascular disease, acute kidney injury, nephrolithiasis, urinary stent, and Charlson comorbidity index), the significant difference in the postimplementation group persisted (odds ratio [OR], 0.42; 95% confidence interval [CI], 0.2–0.70; *P* < .01).

## Discussion

As ASPs continue to evolve and become central to promoting appropriate antimicrobial use, increased emphasis has been placed on enhancing microbiology reporting to influence prescribing and to supplement active ASP efforts. Culture or organism-specific comments serve as a passive mode of provider education. Wording of such comments, when concise and informative, allows for the evolution of noninvasive stewardship when direct contact is not required to influence prescribing, and it likely provides a durable education mechanism.

To our knowledge, this is the first study to specifically evaluate the effect of templated comments for hospitalized patients with candiduria. We detected a 14.1% decrease in antifungal prescribing for patients with candiduria by leveraging passive microbiology comments, further supporting a growing body of research for embedding templated microbiology comments as a useful strategy to improve antimicrobial stewardship. Once a culture shows growth, there may be a natural inclination to treat (if not already); therefore, we targeted antifungal administration within 72 hours of the results becoming available, allowing providers ample time to evaluate culture results and determine need for treatment. Furthermore, the net effect of the comment(s) may be greater; we evaluated their effect in the period immediately after implementation. Despite a few baseline characteristics being higher in the postimplementation group, the primary outcome remained significant even after adjusting for select differences. Importantly, the comparator groups represented the same calendar periods to minimize any seasonal variance or other unintended confounders.

We did not detect any differences in secondary outcomes between groups. For patients receiving antifungal treatment, there was not a significant difference in antifungal duration, though the postimplementation group had 1 less day of exposure. Regarding concerns for the need to add antifungal therapy later in a case when the templated comment incorrectly nudged providers, we found no statistical difference in antifungal administration rates 73–240 hours after cultures resulted. This finding suggests that the comment did not increase the risk of inappropriate treatment decisions secondary to the nudge.

Preceding work in this area has influenced a trend to incorporate templated comments as a stewardship tactic. In a quasi-experimental study, Musgrove et al^3^ evaluated the effect of comments on respiratory cultures. For cultures that grew only normal respiratory flora, they added comments regarding the absence of *Pseudomonas aeruginosa* or *Staphylococcus aureus*, resulting in a higher rate of broad-spectrum antibiotic de-escalation (39% vs 73%; *P* < .01) and a 5.5-fold increased odds of antibiotic de-escalation for pneumonia after adjusting for severity of illness and baseline comorbidities.^3^ The same group recently published a follow-up study demonstrating that the intervention resulted in a durable stewardship practice.^17^ A review by Langford et al^8^ highlighted a more global assessment of various nudging tactics that which can be used to influence antimicrobial prescribing for a number of infectious sources, including urinary. We focused on a different subset of patients, those with urinary growth of *Candida*, which is frequently associated with colonization rather than infection. Similar to previous studies, we also detected a significant reduction in antifungal prescribing using passive culture-embedded guidance. Cumulatively, the works by Musgrove et al,^3^ our study results, and previous work regarding nudging principles support the use of templated comments as an effective antimicrobial stewardship tool for a variety of infectious diseases.

This study had several limitations. First, as previously highlighted, we evaluated the intervention immediately after implementation, which may have minimized the true effect of the templated comments without a phase-in adoption period. A lag period in the postintervention group analysis to allow for providers to become acclimated to practice changes may be important in future study designs. Another limitation is the reliance on manual templated comment entry by microbiology laboratory technicians, which may have been a source of inconsistency in the postintervention group. Nevertheless, the postimplementation group contained all patients with candiduria, regardless of comment placement, to ensure consistency among groups. Since these data were collected, the study site has shifted to automating the addition of templated comments by the laboratory software, a process we endorse to ensure consistency for those wishing to replicate this practice. Importantly, this comment exists for both inpatient and outpatient cultures with *Candida* growth, representing an unassessed cohort who may also benefit from such stewardship interventions. Another limitation was the evaluation of symptomatic disease. Our goal was to evaluate the effect of the intervention on antifungal exposure; therefore, symptomatic versus asymptomatic candiduria was not assessed. Next, the secondary outcome of administration of antifungal use within 73–240 hours is not a validated method to assess safety but represents a window of time when treatment would be initiated should patients decline when treatment was initially withheld. Also, we did not evaluate the need for treatment at the time of microbiology results or the following 30 days after the initial culture results, representing an opportunity for future studies. However, only 1 patient from the postimplementation group had a second urine culture drawn that grew *Candida* and was treated with an antifungal the day after the result. Also, baseline rates of acute kidney injuries were higher in the postimplementation group, which may have resulted in more patients being treated due to increased concerns for active infection. Despite this increase, the primary outcome remained significantly different when controlling for this along with other significant baseline differences. Finally, although an interrupted time-series design could have been considered, we evaluated the practice change during the same period, 1 year apart, and immediately after implementation. Additionally, no other ASP efforts were undertaken to identify these patients; thus, the quasi-experimental design should have accurately characterized practice change given the large (14.1%) change in a single year.

Our study results provide further evidence supporting the use of templated microbiology comments to improve antimicrobial stewardship. These results also show that candiduria is another important target for passive improvement of responsible antimicrobial prescribing and that using templated comments in urine culture microbiology results lead to reduced antifungal exposure.

## References

[1] Barlam T , Cosgrove S , Abbo L , et al. Implementing an antibiotic stewardship program: guidelines by the Infectious Diseases Society of America and the Society for Healthcare Epidemiology of America. Clin Infect Dis 2016;62. 10.1093/cid/ciw118 PMC5006285 27080992

[2] Dellit T , Owens R , McGowan J Jr , et al. Infectious Diseases Society of America and the Society for Healthcare Epidemiology of America guidelines for developing an institutional program to enhance antimicrobial stewardship. Clin Infect Dis 2007;44:159–177. 17173212 10.1086/510393

[3] Musgrove M , Kenney R , Kendall R , et al. Microbiology comment nudge improves pneumonia prescribing. IDSA 2018;5:1–5. 10.1093/ofid/ofy162 PMC6057519 30057928

[4] Meeker D , Linder J , Fox C , et al. Effect of behavioral interventions on inappropriate antibiotic prescribing among primary care. JAMA 2016;315:562–570. 26864410 10.1001/jama.2016.0275 PMC6689234

[5] Gonzales R , Anderer T , McCulloch C , et al. A cluster randomized trial of decision support for reducing antibiotic use in acute bronchitis. JAMA Intern Med 2013;173:267–273. 23319069 10.1001/jamainternmed.2013.1589 PMC3582762

[6] Cunney R , Smyth E. The impact of laboratory reporting practice on antibiotic utilization. Int J Antimicrob Agents 2000;14:13–19. 10717496 10.1016/s0924-8579(99)00144-2

[7] Grayson M , Melvani S , Kirsa S , et al. Impact of an electronic antibiotic advice and approval system on antibiotic prescribing in an Australian teaching hospital. Med J Aust 2004;180:455–458. 15115423 10.5694/j.1326-5377.2004.tb06022.x

[8] Langford B , Leung E , Haj R , et al. Nudging in microbiology laboratory evaluation (NIMBLE): A scoping review. Infect Control Hosp Epidemiol 2019;40:1400–1406. 31679535 10.1017/ice.2019.293

[9] Mcbride J , Schulz L , Fox B , Dipoto J , Sippel N , Osterby K. Influence of a “no MRSA, no *Pseudomonas*” comment to a respiratory culture on antibiotic utilization during the treatment of lower respiratory tract infection. *Open Forum Infect Dis* 2015. doi: 10.1093/ofid/ofy162.

[10] Cortegiani A , Misseri G , Giarratano A , Bassetti M , Eyre D. The global challenge of *Candida auris* in the intensive care unit. Crit Care 2019;23:150. 31046836 10.1186/s13054-019-2449-y PMC6495550

[11] Munoz P , Bouza E , The current treatment landscape: The need for antifungal stewardship programmes. J Antimicrob Chemother 2016;71:5–12. 10.1093/jac/dkw391 27880664

[12] Sobel J , Fisher J , Kauffman C , Newman C. *Candida* urinary tract infections—epidemiology. Clin Infect Dis 2011;52:S433–S436. 21498836 10.1093/cid/cir109

[13] Pappas P , Kauffman C , Andes D , et al. Clinical practice guideline for the management of candidiasis: 2016 update by the Infectious Diseases Society of America. Clin Infect Dis 2016;62:1–50. 26679628 10.1093/cid/civ933 PMC4725385

[14] Munoz P , Valerio M , Vena A , Bouza E. Antifungal stewardship in daily practice and health economic implications. Mycoses 2015;58:14–25. 26033252 10.1111/myc.12329

[15] Jacobs D , Dilworth T , Beyda N , Casapao A , Bowers D. Overtreatment of asymptomatic candiduria among hospitalized patients: a multi-institutional study. Antimicrob Agents Chemother 2018;62:1–8. 10.1128/AAC.01464-17 PMC5740329 29109159

[16] Radosevich J , Nix D , Erstad B. Evaluation of the treatment of candiduria at an academic medical center. Am J Ther 2016;23:e1774–e1780. 26938759 10.1097/MJT.0000000000000021

[17] Kaur S , Hutton M , Kenny R , et al. The long-term sustainability of a respiratory culture nudge. Antimicrob Steward Healthc Epidemiol 2022;2:e15. 36310803 10.1017/ash.2022.5 PMC9614778

